# Extracts from the Mediterranean Food Plants *Carthamus lanatus*, *Cichorium intybus*, and *Cichorium spinosum* Enhanced GSH Levels and Increased Nrf2 Expression in Human Endothelial Cells

**DOI:** 10.1155/2018/6594101

**Published:** 2018-11-15

**Authors:** Dimitrios Stagos, Dimitrios Balabanos, Salomi Savva, Zoi Skaperda, Alexandros Priftis, Efthalia Kerasioti, Eleni V. Mikropoulou, Konstantina Vougogiannopoulou, Sofia Mitakou, Maria Halabalaki, Demetrios Kouretas

**Affiliations:** ^1^Department of Biochemistry and Biotechnology, University of Thessaly, Viopolis, Larissa 41500, Greece; ^2^Division of Pharmacognosy and Natural Product Chemistry, Department of Pharmacy, University of Athens, Panepistimiopolis, Athens 15771, Greece

## Abstract

The Mediterranean diet is considered to prevent several diseases. In the present study, the antioxidant properties of six extracts from Mediterranean plant foods were assessed. The extracts' chemical composition analysis showed that the total polyphenolic content ranged from 56 to 408 GAE mg/g dw of extract. The major polyphenols identified in the extracts were quercetin, luteolin, caftaric acid, caffeoylquinic acid isomers, and cichoric acid. The extracts showed *in vitro* high scavenging potency against ABTS^•+^ and O_2_^•−^ radicals and reducing power activity. Also, the extracts inhibited peroxyl radical-induced cleavage of DNA plasmids. The three most potent extracts, *Cichorium intybus*, *Carthamus lanatus*, and *Cichorium spinosum*, inhibited OH^•^-induced mutations in *Salmonella typhimurium* TA102 cells. Moreover, *C. intybus*, *C. lanatus*, and *C. spinosum* extracts increased the antioxidant molecule glutathione (GSH) by 33.4, 21.5, and 10.5% at 50 *μ*g/ml, respectively, in human endothelial EA.hy926 cells. *C. intybus* extract was also shown to induce in endothelial cells the transcriptional expression of Nrf2 (the major transcription factor of antioxidant genes), as well as of antioxidant genes *GCLC*, *GSR*, *NQO1*, and *HMOX1*. In conclusion, the results suggested that extracts from edible plants may prevent diseases associated especially with endothelium damage.

## 1. Introduction

Reactive oxygen species (ROS) are generated within living organisms by different physiological processes such as metabolism and inflammation [1, 2]. Although basic levels of ROS are needed for cellular homeostasis, they can be harmful when they are overproduced, a condition called oxidative stress [1, 2]. An excessive production of ROS intracellularly may induce oxidative damage to important biological macromolecules [3]. Thus, oxidative stress may be the aetiological factor for a number of pathological conditions, such as cancer, neurodegenerative diseases, diabetes, and cardiovascular diseases [4, 5].

Especially, oxidative stress-induced damage of the vascular endothelium is considered a major cause of cardiovascular ailments [6–8]. For instance, oxidative stress may induce acute and chronic phases of leukocyte adhesion to the endothelium [6, 9]. Moreover, the interplay between ROS and nitric oxide induces a vicious circle that may cause further endothelial activation and inflammation [6, 7]. Furthermore, ROS like hydrogen peroxide (H_2_O_2_) may enter into endothelial cells and interact with cysteine groups in proteins to alter their function [6, 10]. Consequently, oxidative stress may induce different abnormalities to endothelial cells such as progress to senescence loss of integrity and detach into the circulation [11].

Living organisms produce antioxidant molecules, enzymatic and nonenzymatic, for protection against oxidative stress [1, 3]. Moreover, an organism may also obtain antioxidant compounds through diet, especially from plant foods [12, 13]. The antioxidant properties of plant foods are mainly attributed to polyphenols, a large group of secondary metabolites acting as free radical scavengers and metal chelators and affecting the activity of antioxidant enzymes [14]. Consumption of plant products is of great importance in the Mediterranean diet known for its benefits on human health [15]. For example, wild and semidomesticated edible plants containing high polyphenolic content and exhibiting strong antioxidant activity form a major part of the Mediterranean diet [16–20]. Specifically, in Greece, the term “chórta” means wild or semidomesticated edible herbaceous plants, which are cooked or consumed as raw salads as part of the Mediterranean-style Greek diet [16, 17, 21–23]. Currently, there have been few studies on the antioxidant activity of wild edible greens of Greece and especially on the molecular mechanisms accounting for this activity. In a recent preliminary study, we have found that extracts from “chórta” species possessed anticarcinogenic and antioxidant potential [24].

Therefore, the aim of the present study was a further investigation of the antioxidant properties of extracts derived from six wild edible greens (i.e., *Carthamus lanatus*, *Crepis sancta*, *Cichorium intybus*, *Cichorium spinosum*, *Amaranthus blitum*, and *Sonchus asper*) from Greece. Thus, the extracts were examined for their free radical scavenging activity against the ABTS^•+^ radical and superoxide anion radical (O2^•−^), for their reducing power activity and for their antimutagenic activity against ROS-induced mutagenicity. Moreover, the extracts' possible enhancement of antioxidant defense in endothelial cells and the molecular mechanisms accounting for these effects was investigated.

## 2. Materials and Methods

### 2.1. Plant Material and Isolation of Extracts

Six plant species, *C. lanatus* (gkourounáki), *C. intybus* (kavouráki), *C. sancta* (ladáki), *S. asper* (zochós), *C. spinosum* (stamnagkáthi), and *A. blitum* (vlíto), were obtained from local markets in Athens (Greece; spring of 2015). Five of them were from the family of Asteraceae and one from the family of Amaranthaceae (i.e., *A. blitum*). The samples were botanically characterized at the Laboratory of Pharmacognosy and Natural Products Chemistry. As described previously [24], the leaves and stems were boiled with water (500 g of plant material/1 l of water), for 20 minutes. After cooling at room temperature, the decoctions were filtered through paper and evaporated to dryness. Moreover, three of the extracts and more specifically those of *C. lanatus*, *C. sancta*, and *C. intybus* were enriched by using XAD7 HP Amberlite® adsorption resin. All of the dry extracts were submitted to HPLC-PDA chemical analysis.

### 2.2. HPLC-PDA Analysis

For the HPLC analysis of the extracts, a Thermo Finnigan® HPLC-PDA System (P4000 Pump, AS3000 Autosampler, PDA Detector UV8000, ChromQuest™ 4.2 Software) and a Supelco® RP18 Discovery HS-C18 (250 mm, 4.6 mm, and 5 *μ*m) column were employed. 20 *μ*l of water extracts at 1.5 mg/ml was injected. The mobile phase was 0.1% formic acid in water (A) and MeOH (B). Elution started with 2% (B), reaching 100% (B) in 60 minutes. These conditions were kept for 4 minutes before getting back to initial conditions in 2 min for a 4 min reequilibration. The flow rate was maintained at 1 ml/min and the column temperature at 25°C. Relative quantification of the main secondary metabolites was performed at 280 nm absorbance.

### 2.3. Evaluation of the Total Polyphenolic Content (TPC) of the Extracts

The evaluation of the TPC of the plant extracts was assessed spectrophotometrically at 765 nm by using the Folin-Ciocalteu reagent as described previously [25]. TPC was determined by a standard curve of absorbance values in correlation with standard concentrations (50–1500 *μ*g/ml) of gallic acid. The TPC was expressed as mg of gallic acid equivalents (GAE) per g of dry weight (dw) of extract.

### 2.4. ABTS^•+^ Radical Scavenging Assay

ABTS^•+^ radical scavenging capacity of the extracts was performed as described previously [26]. Briefly, ABTS^•+^ radical was generated by mixing 2 mM ABTS with 30 *μ*M H_2_O_2_ and 6 *μ*M horseradish peroxidase (HRP) enzyme in 1 ml of distilled water. The mixture was vortexed vigorously and left at room temperature in the dark for 45 min. Subsequently, 10 *μ*l of extract at different concentrations was added in the reaction solution and the absorbance at 730 nm was read. In each experiment, a blank was used consisting of the tested sample in distilled water, ABTS^•+^, and H_2_O_2_. The ABTS^•+^ radical solution with 10 *μ*l H_2_O was used as control. After measuring the absorbance, the percentage of radical scavenging capacity (RSC) of the tested extracts was calculated. In addition, for comparison of the extracts' radical scavenging efficiency, IC_50_ value indicating the concentration that caused 50% scavenging of ABTS^•+^ radical was calculated from the graph-plotted RSC percentage against extract concentration. At least two independent experiments in triplicate were performed for each tested compound.

### 2.5. Superoxide Radical Scavenging Assay

The superoxide anion radical (O_2_^•−^) scavenging activity of the extracts was evaluated as described previously [27]. In brief, O_2_^•^ is produced by the PMS-NADH system through oxidation of NADH and is measured spectrophotometrically at 560 nm by the reduction of nitroblue tetrazolium (NBT). Antioxidants may scavenge O_2_^•−^ and consequently reduce absorbance. The RSC and the IC_50_ values for O_2_^•−^ were evaluated as mentioned above for ABTS^•+^ radical. At least two independent experiments in triplicate were performed for each tested compound.

### 2.6. Reducing Power Assay

Reducing power was determined spectrophotometrically as described previously [28]. RP_0.5AU_ value showing the extract concentration-caused absorbance of 0.5 at 700 nm was calculated from the graph-plotted absorbance against extract concentration. At least two independent experiments in triplicate were performed for each tested compound.

### 2.7. Peroxyl Radical-Induced DNA Plasmid Strand Cleavage

The assay was performed as described previously [29]. In brief, peroxyl radicals (ROO•) were produced from thermal decomposition of 2,2′-azobis (2-amidinopropane hydrochloride) (AAPH). The reaction mixture (10 *μ*l) containing 1 *μ*g pBluescript-SK+ plasmid DNA, 2.5 mM AAPH in phosphate-buffered saline (PBS), and the tested extract at different concentrations was incubated in the dark for 45 min at 37°C. Then the reaction was stopped by the addition of 3 *μ*l loading buffer (0.25% bromophenol blue and 30% glycerol). After analyzing the DNA samples by agarose gel electrophoresis, they were photographed and analyzed using the Alpha Innotech Multi Image (ProteinSimple, California, USA). In addition, plasmid DNA was treated with each extract alone at the highest concentration used in the assay in order to test their effects on plasmid DNA conformation. The percentage of the protective activity of the tested extracts from ROO•-induced DNA strand breakage was calculated using the following formula:

(1) $$\begin{array}{c} \%inhibition=\left[\frac{\left(S–S_{0}\right)}{\left(S_{control}–S_{0}\right)}\right]\times 100, \end{array}$$

where *S*_control_ is the percentage of supercoiled DNA in the negative control sample (plasmid DNA alone), *S*_0_ is the percentage of supercoiled plasmid DNA in the positive control sample (without tested extracts but in the presence of the radical initiating factor), and *S* is the percentage of supercoiled plasmid DNA in the sample with the tested extracts and the radical initiating factor. Moreover, IC_50_ values showing the concentration that inhibited the AAPH-induced relaxation by 50% were calculated from the graph-plotted percentage inhibition against extract concentration. At least two independent experiments in triplicate were performed for each tested compound.

### 2.8. Bacterial Strain

Seven hundred microliters of the stock culture of *Salmonella typhimurium* TA102 strain (MOLTOX, Boone, NC) was used to inoculate 30 ml of Oxoid nutrient broth no. 2. The inoculated cultures were placed on a shaker (100 rpm) and incubated in the dark at 37°C until the cells reached a density of 1-2 × 10^9^ colony forming units (CFU/ml, OD_540_ between 0.1 and 0.2).

### 2.9. The Antimutagenicity Test

Two of the extracts (i.e., *C. lanatus* and *C. intybus*) enriched with polyphenols that exhibited the highest protective activity against ROO^•^-induced DNA plasmid damage were also examined for their inhibitory activity against ROS-induced mutagenicity in *S. typhimurium* TA102 bacterial cells. Similarly, *C. spinosum* extract, the most potent among nonenriched extracts, was also examined for its antimutagenic activity in *S. typhimurium* TA102 bacterial cells.

For the antimutagenicity examination, the standard plate incorporation procedure was used as described previously [27, 30, 31]. *Tert*-butyl hydroperoxide (*t*-BOOH) was used as mutagenic agent. Specifically, the following substances were added in screwed sterile tubes maintained at 45°C ± 2°C: 2 ml top agar, 100 *μ*l bacterial culture of *S. typhimurium* TA102 strain, 50 *μ*l *t*-BOOH solution (0.4 mM final concentration), and 50 *μ*l extract at various concentrations. The contents of the tubes were mixed and poured onto the surface of glucose minimal agar plates. Then the plates were inverted and placed in an incubator, at 37°C ± 2°C for 48 h. Afterwards, the histidine revertant colonies (His^+^) were counted. Before counting, the agar plates were microscopically checked for toxicity [31]. Each assay included both positive (oxidizing agent alone) and negative (plates without oxidizing agent or tested extract) controls. Also, each antioxidant was checked at the two highest concentrations used in the antimutagenicity assay, for possible induction of mutations.

For evaluation of the percent inhibition of mutagenicity, the number of induced revertants was obtained by subtracting the number of spontaneous revertants from the number of revertants on the plates containing the mutagen and/or antioxidant. The percentage inhibition of mutagenicity was calculated as follows:

(2) $$\begin{array}{c} Inhibition=\left[1-\frac{number\text{ of }colonies/plate\;with\;oxidant+test\;compound}{number\text{ of }colonies/plate\;with\;oxidant\;alone}\right]\times 100. \end{array}$$

At least two independent experiments in triplicate were performed for each tested compound.

### 2.10. Cell Culture Conditions

As described previously [32], human endothelial EA.hy926 cells were cultured in normal Dulbecco's modified Eagle's medium (DMEM) in plastic disposable tissue culture flasks at 37°C in 5% carbon dioxide.

### 2.11. XTT Assay

To examine the extracts' antioxidant activity in endothelial cells, noncytotoxic concentrations were used. For selection of these concentrations, extracts' cytotoxicity in endothelial cells was checked using the cell viability XTT assay kit (Roche, Switzerland) as described previously [28]. Briefly, EA.hy926 cells were seeded into a 96-well plate with 1 × 10^4^ cells per well in DMEM medium. After 24 h incubation, the cells were treated with different concentrations of the extracts in serum-free DMEM medium for 24 h. Then 50 *μ*l of XTT test solution was added to each well. After 4 h of incubation, absorbance was measured at 450 nm and also at 630 nm as a reference wavelength in a Biotek ELx800 microplate reader (Winooski, Vermont, USA). Negative control was DMEM serum-free medium. The absorbance values of the control and samples were used for calculation of the percentage inhibition of cell growth caused by the extract treatment. All experiments were carried out in triplicate and on two separate occasions.

### 2.12. Treatment of EA.hy926 Cells with the Extracts

*C. lanatus* and *C. intybus* extracts which exhibited the highest free radical scavenging potency among extracts enriched with polyphenols were examined for their antioxidant capacity in endothelial EA.hy926 cells. *C. spinosum* extract, the most potent of nonenriched with polyphenols extracts, was also examined in endothelial cells. The cells were cultured in flasks for 24 h. Afterwards, the medium was replaced with serum-free medium containing the tested extracts at noncytotoxic concentrations. The cells were treated with the extracts for 24 h, and then they were trypsinized, collected, and centrifuged twice at 300 × g for 10 min at 5°C. At the end of the first centrifugation, the supernatant fluid was discarded and the cellular pellet was resuspended in PBS. After the second centrifugation, the cell pellet was collected and used to measure the glutathione (GSH) and ROS levels and the mRNA levels of antioxidant genes.

### 2.13. Assessment of GSH and ROS Levels by Flow Cytometry Analysis in Endothelial Cells

The GSH and ROS levels in EA.hy926 cells were assessed using mercury orange and DCF-DA, respectively, as described previously [33, 34]. In brief, for assessment of the GSH and ROS levels, the cells were resuspended in PBS at 1 × 10^6^ cells/ml and incubated in the presence of mercury orange (10 *μ*Μ) and DCF-DA (40 *μ*Μ), respectively, in the dark at 37°C for 30 min. Then the cells were washed, resuspended in PBS, and submitted to flow cytometric analysis using a FACSCalibur flow cytometer (Becton Dickinson, New Jersey, USA) with excitation and emission wavelengths at 488 and 530 nm for ROS and at 488 and 580 nm for GSH. Data were analyzed using “BD CellQuest” software (Becton Dickinson). Each experiment was repeated at least three times.

### 2.14. Quantitative Real-Time PCR (qRT-PCR) of Antioxidant Genes

The extract that exhibited the highest antioxidant potency in endothelial cells (i.e., *C. intybus*) was examined for its effects on the transcriptional expression of major antioxidant genes, as described previously [35]. Specifically, EA.hy926 cells were treated with *C. intybus* extract at 50 *μ*g/ml for 3, 12, and 24 h. Then RNA was extracted from cell pellet (see Section 2.11) using an RNA isolation kit (PureLink™ RNA kit, Invitrogen, USA). The extracted RNA (~10 *μ*g) was treated with DNase (RQ1 RNase-Free DNase, 1 U/*μ*l, Promega, USA). DNA-free RNA was then reverse transcribed to obtain cDNA (SuperScript II Reverse Transcriptase, Invitrogen, USA) using oligo (dT) 12-18 primers (Invitrogen, USA). Amplification of cDNAs for the *NFE2L2*, *GCLC*, *GSR*, *GPX1*, *HMOX1*, *CAT*, *SOD1*, *NQO1*, *TXN*, and *GAPDH* genes was carried out in 10 *μ*l reactions containing SYBR® Select Master Mix 2X (Applied Biosystems, CA, USA), 0.25 *μ*Μ of each primer, 50 nM ROX Low, and 25 ng cDNA for the amplification of all genes. The utilized primers are shown in Table 1. The thermocycling conditions used for the amplification of the aforementioned genes were the following: 3 min at 95°C, 45 cycles of 15 sec at 95°C, and 30 sec at 53°C followed by 30 sec at 72°C. Finally, a melting curve was carried out from 53°C to 95°C to check the specificity of the products. All qRT-PCR were performed on a *μ*x3005P system (Stratagene, UK). Amplification efficiencies were >86% with *r* ^2^ values > 0.981 for all genes.

### 2.15. Statistical Analysis

All results were expressed as mean ± SD. Differences were considered significant at *p* < 0.05. One-way ANOVA was performed followed by Tukey's test for multiple pair-wise comparisons using the SPSS 20.0 software.

## 3. Results and Discussion

### 3.1. Polyphenolic Composition of Extracts

**Figure 1:**
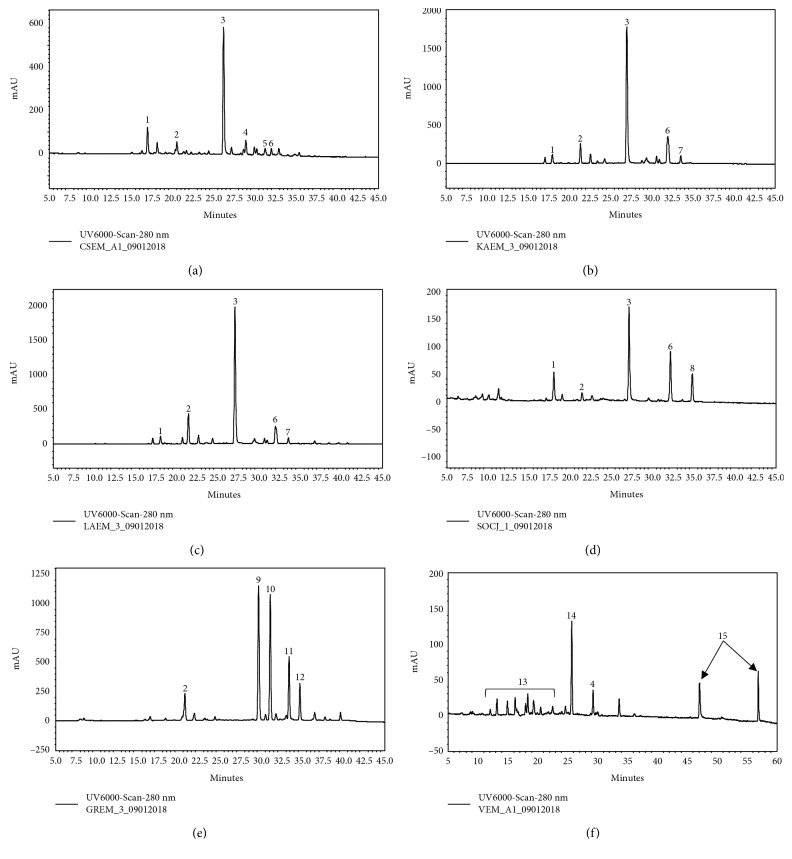
HPLC-PDA profiles of plant decoctions: (a) *Cichorium spinosum*, (b) *Cichorium intybus*, (c) *Crepis sancta*, (d) *Sonchus asper*, (e) *Carthamus lanatus*, (f) *Amaranthus blitum*, and their main metabolites: 1: caftaric acid; 2: caffeoylquinic acid isomer; 3: cichoric acid; 4: luteolin diglycoside; 5: quercetin glucuronide; 6: luteolin glucuronide; 7: dicaffeoylquinic acid isomer; 8: apigenin glucuronide; 9: quercetin glucoside; 10: luteolin glucoside; 11: quercetin acetyl glucoside; 12: luteolin acetyl glucoside; 13: hydroxycinnamates; 14: rutin; 15: triterpene saponins.

The range of the TPC in the tested extracts was from 56 to 408 mg GAE/gr dw (Table 2). As expected, *C. lanatus*, *C. intybus*, and *C. sancta* exhibited the higher TPC values (408, 320, and 288 mg GAE/gr dw of extract, respectively), since they were enriched with polyphenols by using absorption resin (Table 2). All the extracts from the Asteraceae family were rich in phenolic compounds and particularly hydroxycinnamic acids and flavonoid derivatives. All the members of the Cichoriae tribe (i.e., *C. spinosum*, *C. intybus*, *C. sancta*, and *S. asper*) presented a similar chemical profile (Figure 1) with variations to the relative percentages of specific secondary metabolites in each extract (supplementary material (available here)). Based on the literature [36] and previous LC-MS analyses (data not shown) of the extracts, we estimated that two caffeoyl tartaric acid derivatives, namely, caftaric acid and cichoric acid, constituted the predominant compounds of these extracts and could be responsible for the decoctions' biological activities. *C. lanatus*-enriched decoction appeared to possess extremely high amounts of phenolic substances, most probably glycosides of the flavonoids quercetin and luteolin, while its profile was complimented by the presence of caffeoylquinic acid isomers and dimers. On the other hand, the *A. blitum* extract was quite poor in phenolic compounds with only a few, minor peaks observed in the medium polarity area of its chromatogram. However, as expected from a member of the Amaranthaceae family, the presence of the nonpolar triterpene saponins was evident.

### 3.2. Free Radical Scavenging Activity and Reducing Power of Extracts

The IC_50_ values against ABTS^•+^ and O_2_^•−^ radicals are shown in Table 2. Low IC_50_ values mean strong antioxidant potency. In the ABTS^•+^ method, the IC_50_ extended from 7.9 (*C. lanatus*) to 72.0 *μ*g/ml (*A. blitum*), and in O_2_^•−^ radical assay, it was from 6.3 (*C. lanatus*) to 56.0 *μ*g/ml (*S. asper*) (Table 2). It was remarkable that *C. lanatus* extract had the highest potency in ABTS^•+^ and O_2_^•−^ radical assays and exhibited activity at too low concentrations. As mentioned, *C. lanatus* had also the highest TPC, and so, its rich polyphenolic content may explain the high antioxidant activity. Moreover, the polyphenols (i.e., quercetin, luteolin, and caffeoylquinic acid derivatives) found in *C. lanatus* have been known as strong free radical scavengers [37, 38]. In general, the three extracts (i.e., *C. lanatus*, *C. intybus*, and *C. sancta*) enriched with polyphenols due to use of absorption resin exhibited IC_50_ values which were at least 2-fold lower than those of the other extracts (Table 2). Although ABTS^•+^ radical is one of the most used for examining compounds' antioxidant activity, it is a synthetic radical. However, O_2_^•−^ is one of the most common and reactive radicals found in living organisms [3]. Superoxide radical may be produced *in vivo* by the reactions of the electron transport chains in mitochondria (1–3% of electrons forming O_2_^•−^), activated phagocytic cells, enzymatic activity (e.g., P450 enzymes and xanthine oxidase), and autooxidation reactions of biomolecules (e.g., adrenalin and FADH_2_) [2]. Superoxide radical may cause damage to DNA, proteins, and lipids, and these effects seem to increase with aging [3]. Thus, it is important for prevention of oxidative stress-induced diseases to find out compounds which effectively scavenge O_2_^•−^.

In the reducing power assay, RP_0.5AU_ values ranged from 5.0 (*C. lanatus*) to 65.0 (*A. blitum*) (Table 2). Like IC_50_ values, the lower the RP_0.5AU_ value is, the higher is the reducing power activity. The reducing power of a compound is an indication of its antioxidant activity, because it shows its ability to act as an electron donor and consequently to neutralize free radicals [39]. Again, *C. lanatus* extract was the most potent in this assay, while the other two extracts (i.e., *C. intybus* and *C. sancta*) that were enriched with polyphenols had also high reducing activity (Table 2). However, in this assay, *C. spinosum* extract also exhibited high reducing potency (RP_0.5AU_: 8.0), although it was not processed with absorption resins (Table 2). This *C. spinosum* extract's high activity may be explained by the type of its polyphenols or by the presence of other chemical compounds which may be very effective as hydrogen donors.

### 3.3. Antimutagenic Activity of Extracts against ROS-Induced DNA Damage

**Figure 2:**
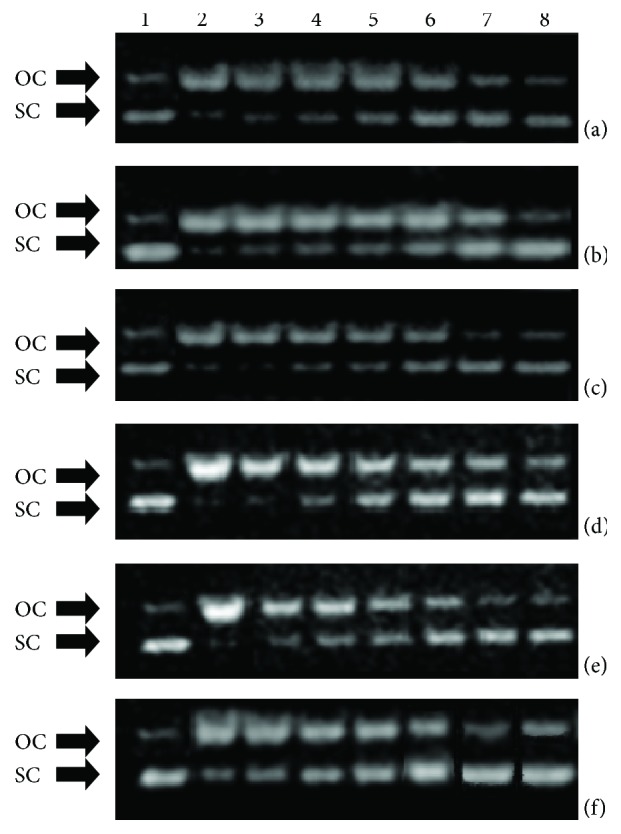
Protective activity of extracts from (a) *Crepis sancta*, (b) *Cichorium intybus*, (c) *Carthamus lanatus*, (d) *Amaranthus blitum*, (e) *Cichorium spinosum*, and (f) *Sonchus asper* plants against ROO^•^ radicals: 1: pBluescript-SK+ plasmid DNA without any treatment; 2: plasmid DNA exposed to ROO^•^ radicals alone; 3, 4, 5, 6, and 7: plasmid DNA exposed to ROO^•^ radicals in the presence of extract concentrations: 0.015, 0.030, 0.060, 0.120, and 0.240 mg/mL, respectively (a–c) and 0.030, 0.060, 0.120, 0.240, and 0.480 mg/ml, respectively (e) and 0.120, 0.240, 0.480, 0.960, and 1.920 mg/ml, respectively (d, f); 8: plasmid DNA exposed to the maximum tested concentration of each extract alone. OC: open circular; SC: supercoiled.

All the tested extracts inhibited ROO•-induced DNA plasmid breakage, with IC_50_ values ranging from 105 to 970 *μ*g/ml (Table 2 and Figure 2). The potency order was *C. lanatus* = *C. intybus* > *C. sancta* > *C. spinosum* > *A. blitum* > *S. asper* (Table 2). Similar to antioxidant assays, the three extracts enriched with polyphenols by passage through resin column exhibited at least 2-fold higher protective activity compared to other extracts. The ROO• radicals that caused the DNA damage in this assay are produced within cells after the addition of O_2_ to carbon-centered radicals [40]. Subsequently, ROO• can oxidize DNA bases to their hydroxyl derivatives resulting in mutations and manifestation of diseases [41]. These detrimental effects may be prevented by the use of the tested extracts through diet as the present findings suggested. Interestingly, quercetin, luteolin, and caffeoylquinic acid found in *C. lanatus* extract have been demonstrated to scavenge ROO• [42, 43].

**Figure 3:**
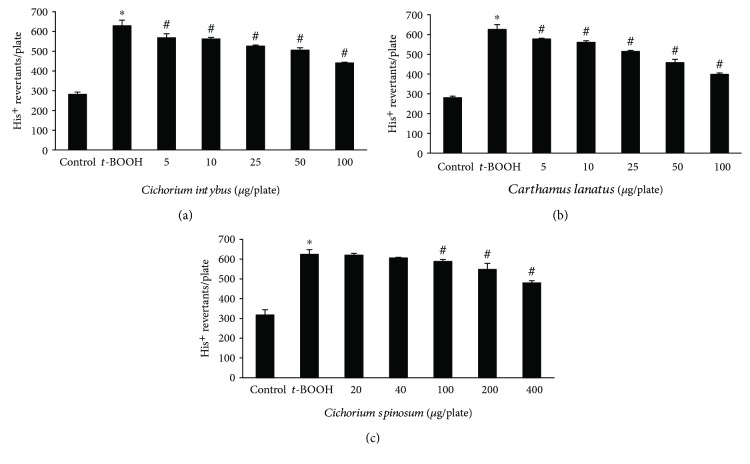
Antimutagenic effects of *C. intybus*, *C. lanatus*, and *C. spinosum* extracts on *t*-BOOH-induced mutagenicity in *S. typhimurium* TA102 cells. Values are the mean ± SD number of histidine revertants of three independent experiments carried out in triplicate. The concentration of *t*-BOOH was 0.4 mM/plate. ^∗^ *p* < 0.05 when compared with control. ^#^ *p* < 0.05 when compared with the *t*-BOOH alone sample.

The two most potent extracts enriched with polyphenols due to passage through resin (i.e., *C. lanatus* and *C. intybus*) and the most potent (i.e., *C. spinosum*) among nonenriched extracts were also tested for their inhibitory potency against ROS-induced mutagenicity in *S. typhimurium* TA102 bacterial cells. The results from this assay supported those from DNA plasmid cleavage assay, since all three extracts inhibited dose-dependent *t*-BOOH-induced mutagenicity (Figure 3). Specifically, *C. lanatus* extract inhibited significantly *t*-BOOH-induced mutations by 12.3, 17.9, 31.7, 48.7, and 66.9% at 5, 10, 25, 50, and 100 *μ*g/plate, respectively, *C. intybus* extract by 17.0, 19.6, 30.0, 38.0, and 56.9% at 5, 10, 25, 50, and 100 *μ*g/plate, respectively, and *C. spinosum* extract by 12.2, 24.7, and 48.1% at 100, 200, and 400 *μ*g/plate, respectively (Figure 3). The two extracts enriched with polyphenols had higher inhibitory activity than the nonenriched extract, indicating that the polyphenols accounted mainly for the observed protection from mutagenicity. The *t*-BOOH reacts with Fe^2+^ in cells and generates HO• causing DNA damage [3]. The OH• radicals are the major ROS to react with either DNA bases or deoxyribose resulting in DNA damage and mutations [3]. Thus, it was important that the tested extracts protected from OH•-induced DNA mutations. *C. lanatus* extract was again the most potent due, at least in part, to its identified polyphenols, since previous studies have shown that quercetin and luteolin inhibited *t*-BOOH-induced mutagenicity in TA102 cells [44]. Moreover, Jho et al. [45] have reported that a caffeoylquinic derivative inhibited *t*-BOOH-induced DNA damage in human liver HepG2 cells.

### 3.4. Effects of Extracts on the Antioxidant Status of Endothelial Cells

**Figure 4:**
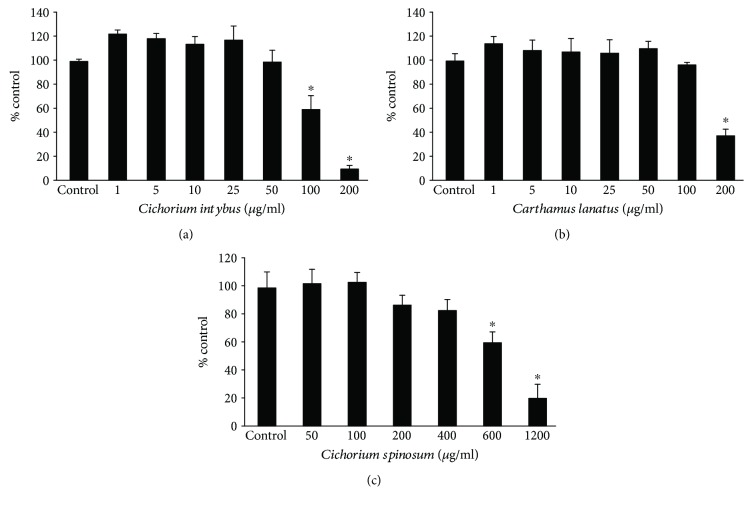
Cell viability following treatment with *C. intybus*, *C. lanatus*, and *C. spinosum* extracts in EA.hy926 cells. The results are presented as the means ± SEM of three independent experiments carried out in triplicate. ^∗^ *p* < 0.05 indicates significant difference from the control value.

*C. lanatus* and *C. intybus* extracts, the two most potent extracts enriched with polyphenols, and *C. spinosum* extract, the most potent among nonenriched extracts, were examined for their antioxidant activity in human endothelial EA.hy926 cells. Firstly, the extracts' cytotoxicity was evaluated, so as noncytotoxic concentrations to be used for the assessment of the antioxidant activity. The results from XTT assay showed that *C. intybus*, *C. lanatus*, and *C. spinosum* extracts exhibited significant cytotoxicity above 100, 200, and 600 *μ*g/ml, respectively (Figure 4). Thus, the selected concentrations of the *C. intybus*, *C. lanatus*, and *C. spinosum* extracts in the following assays were up to 50, 100, and 400 *μ*g/ml, respectively.

**Figure 5:**
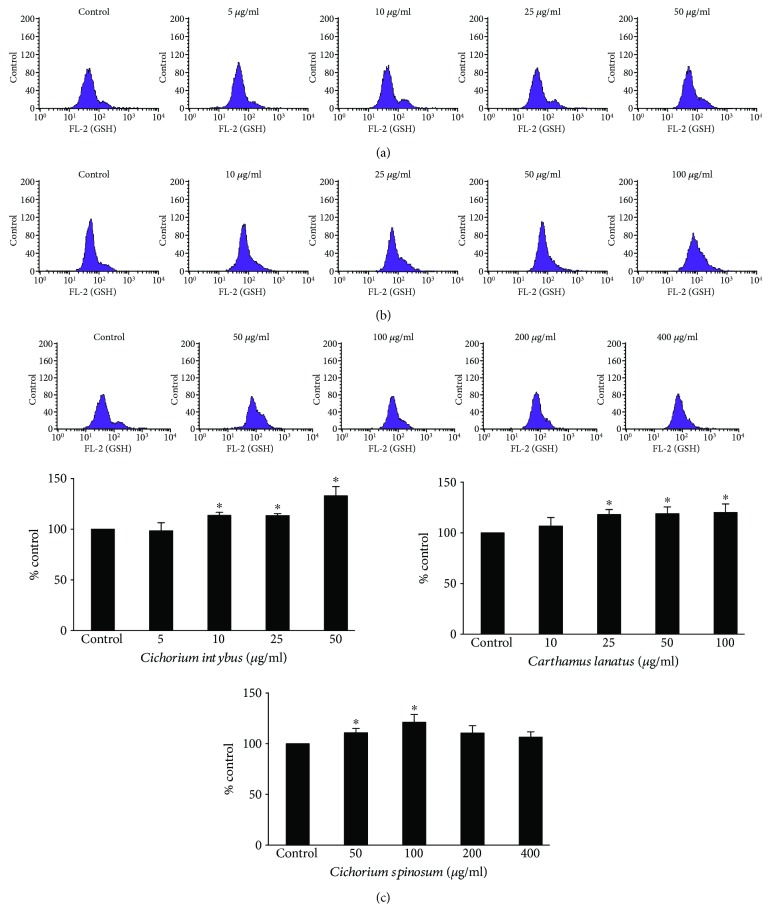
Effects of treatment with *C. intybus*, *C. lanatus*, and *C. spinosum* extracts at different concentrations for 24 h on GSH levels in EA.hy926 cells. The histograms of cell counts versus fluorescence of 10,000 cells analyzed by flow cytometry for the detection of GSH levels after treatment with (a) *C. intybus*, (b) *C. lanatus*, and (c) *C. spinosum*. FL2 represents the detection of fluorescence using 488 and 580 nm as the excitation and emission wavelength, respectively. Bar charts indicate the GSH levels as % of control as estimated by the histograms in EA.hy926 cells after treatment with *C. intybus*, *C. lanatus*, and *C. spinosum* extracts. All values of bar charts are presented as the mean ± SEM of 3 independent experiments. ^∗^ *p* < 0.05 indicates significant difference from the control.

The assessment of the extracts' effects on the antioxidant capacity of endothelial cells was based on the measurement of GSH and ROS levels by flow cytometry. The results showed that *C. intybus* increased significantly GSH levels by 10.8, 15.2, and 33.4% at 10, 25, and 50 *μ*g/ml, respectively, compared to control, *C. lanatus* by 15.9, 21.5, and 24.7% at 25, 50, and 100 *μ*g/ml, respectively, and *C. spinosum* by 10.5 and 21.6% at 50 and 100 *μ*g/ml, respectively (Figure 5). The increase in GSH after extract treatment is important, since GSH is considered as a significant endogenous antioxidant molecule in cells [46]. GSH may scavenge directly free radicals by donating one hydrogen atom from its sulfhydryl group or is used as substrate by antioxidant enzymes such as glutathione transferase (GST) and glutathione peroxidase (GPx) [46]. Especially for endothelial cells, GSH is important not only as antioxidant but also as a crucial regulator of cell signaling [47, 48]. Although the effects of *C. intybus* and *C. lanatus* on GSH were dose dependent, the *C. spinosum* extract did not affect GSH at higher concentrations than 100 *μ*g/ml (Figure 5). This intriguing observation may be explained by the fact that *C. spinosum* at 200 and 400 *μ*g/ml exhibited a tension to decrease cell viability (Figure 4). That is, for *C. spinosum*, 100 *μ*g/ml seemed to be a crucial concentration, above which cytotoxicity was caused. In turn, this cytotoxicity was encountered by GSH consumption. It is known that polyphenols sometimes have a biphasic effect, namely, at low concentrations, they act as antioxidants and at high concentrations, they act as prooxidants resulting in cytotoxicity [49]. It is also worth mentioning that polyphenols identified in the tested extracts have been reported to increase GSH levels. For example, quercetin has been demonstrated recently to enhance GSH levels in human aortic endothelial cells (HAEC) through increased expression of glutamate-cysteine ligase (GCL), one of the major enzymes involved in GSH synthesis [50]. Moreover, luteolin has been demonstrated to decrease oxidative stress in the mouse lung through, among other mechanisms, increase of GSH [51]. Additionally, administration of caftaric acid to rats inhibited lead-induced decrease in GSH in the kidney [52].

**Figure 6:**
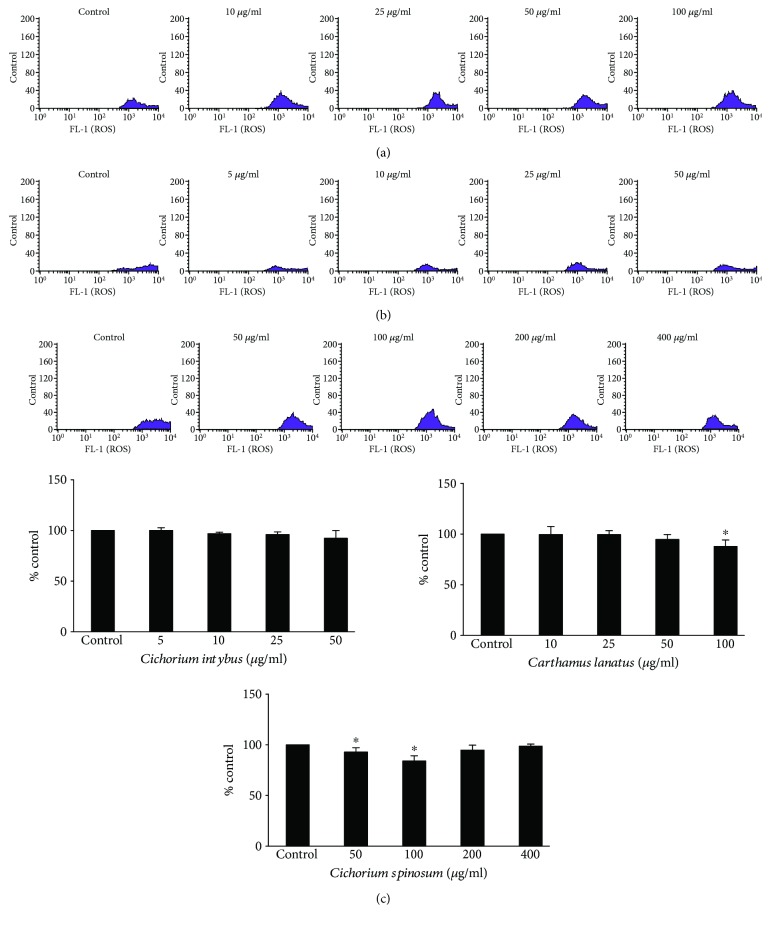
Effects of treatment with *C. intybus*, *C. lanatus*, and *C. spinosum* extracts at different concentrations for 24 h on ROS levels in EA.hy926 cells. The histograms of cell counts versus fluorescence of 10,000 cells analyzed by flow cytometry for the detection of ROS levels after treatment with (a) *C. intybus*, (b) *C. lanatus*, and (c) *C. spinosum*. FL2 represents the detection of fluorescence using 488 and 530 nm as the excitation and emission wavelength, respectively. Bar charts indicate the ROS levels as % of control as estimated by the histograms in EA.hy926 cells after treatment with *C. intybus*, *C. lanatus*, and *C. spinosum* extracts. All values of bar charts are presented as the mean ± SEM of 3 independent experiments. ^∗^ *p* < 0.05 indicates significant difference from the control.

Unlike GSH, extract treatment did not affect ROS levels too intensively (Figure 6). Only *C. lanatus* extract reduced significantly ROS by 12.1% and *C. spinosum* extract by 6.8 and 15.6% at 50 and 100 *μ*g/ml, respectively, compared to control (Figure 6). The weak extracts' effect on ROS levels may be attributed to the fact that their impact was examined on the baseline ROS levels, that is, there was not an oxidant stimulus to cells. Nevertheless, the observed decrease in ROS, even only at the higher extract concentrations, was in accordance with and might be attributed to the corresponding increase in the antioxidant molecule of GSH by the extracts (Figure 5). Interestingly, *C. spinosum* at concentrations higher than 100 *μ*g/ml did not decrease further ROS levels (Figure 6). This finding supported our hypothesis mentioned above, that is, *C. spinosum* at concentrations above 100 *μ*g/ml may exhibit prooxidant effect and cytotoxicity.

**Figure 7:**
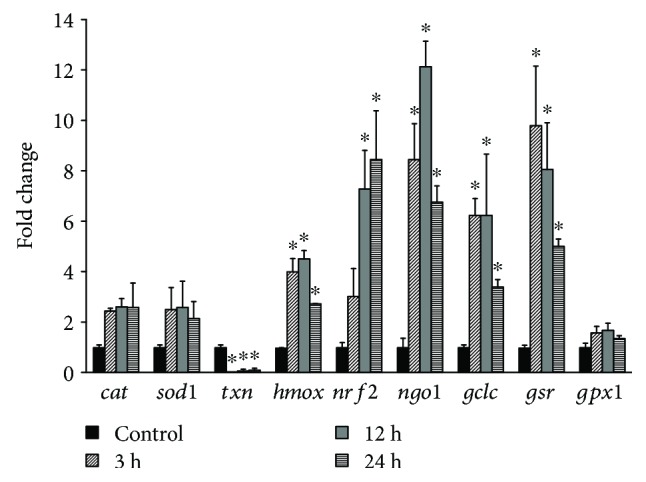
Gene expression profiles of *NFE2L2*, *GCLC*, *GSR*, *GPX1*, *HMOX1*, *NQO1*, *CAT*, *SOD1*, and *TXN* genes in EA.hy926 cells after treatment with *C. intybus* extract at 50 *μ*g/ml for 3, 12, and 24 h. mRNA levels were determined by qRT-PCR, and relative levels were expressed as fold of control (untreated cells) after normalization to GAPDH. The results are expressed as mean ± SD of three independent experiments. ^∗^ *p* < 0.05 indicates significant difference from the control.

Since *C. intybus* extract induced the highest increase in antioxidant mechanism (i.e., GSH) in endothelial cells, its effects on the expression at a transcriptional level of antioxidant genes were assessed (Figure 7). Specifically, it was examined if the extract affected the expression of the nuclear factor (erythroid-derived 2)-like 2 (Nrf2), the most important transcription factor regulating antioxidant genes [53]. The results from the qRT-PCR showed that *C. intybus* treatment upregulated significantly the expression of *NFE2L2* gene encoding for Nrf2 by 7.3-fold and 8.5-fold at 12 and 24 h, respectively, compared to control (Figure 7). This finding was significant, because it indicated that the extract's compounds exerted antioxidant activity not only as direct free radical scavengers but also as modulators of molecular mechanisms. Interestingly, chicoric acid found in *C. intybus* extract has been reported to increase Nrf2 expression in mouse muscle [54]. The increase in Nrf2 expression was supported by the extract treatment-induced increase in expression of genes regulated by Nrf2. Namely, extract treatment upregulated significantly the expression of *GCLC* by 6.2-fold, 6.3-fold, and 3.4-fold at 3, 12, and 24 h, respectively, *GSR* by 9.8-fold, 8.0-fold, and 5.0-fold at 3, 12, and 24 h, respectively, *NQO1* by 8.4-fold, 12.1-fold, and 6.7-fold at 3, 12, and 24 h, respectively, and *HMOX1* by 4.0-fold, 4.5-fold, and 2.7-fold at 3, 12, and 24 h, respectively, compared to control (Figure 7). Especially, the increase in *GCLC* and *GSR* expression was important, since it accounted for the *C. intybus* extract-induced increase in GSH levels (Figure 5). *GCLC* gene encodes the catalytic subunit of the GCL protein, the main enzyme involved in GSH synthesis [40]. *GSR* encodes for glutathione reductase (GR) enzyme regenerating GSH from the oxidized glutathione (GSSG) [40]. *HMOX1* and *NQO1*, the other two upregulating genes, encode for heme oxygenase 1 (HO-1) and NAD (P)H:quinone oxidoreductase 1 (NQO1), respectively. The increase in the expression of these enzymes supported further the ability of the *C. intybus* extract to enhance endothelial cells' antioxidant capacity, since HO-1 and NQO1 are important antioxidant enzymes participating in iron sequestration and quinone detoxification, respectively [55, 56]. *C. intybus* treatment did not affect the expression of *CAT*, *SOD1*, and *GPX1* genes, while it downregulated significantly *TXN* gene expression by 35.7-fold, 10.3-fold, and 10.0-fold at 3, 12, and 24 h, respectively (Figure 7). The lack of effect on *CAT*, *SOD1*, and *GPX1* and the downregulation of *TXN* gene were intriguing, since Nrf2 activates their expression [53]. This contradiction may be explained by the high complexity of the regulation of the antioxidant mechanisms and the interaction between them, namely, when some antioxidant mechanisms are enhanced, some others remain inactive as a compensation adaptive response of the cell [57].

## 4. Conclusions

In conclusion, the present findings demonstrated for the first time that extracts from the edible plants *C. lanatus*, *C. intybus*, and *C. spinosum* enhanced antioxidant defense mechanism such as GSH in endothelial cells. Especially, *C. spinosum* extract was shown to mediate this antioxidant effect through increased expression of Nrf2, the most crucial transcription factor of antioxidant genes, and subsequent upregulation of important antioxidant genes including those involved in GSH synthesis. Since, these plants constitute a part of the Mediterranean diet, their observed bioactivities may explain, at least in part, the prevention of this type of diet against diseases associated with endothelial function such as the cardiovascular disease [58]. Moreover, the results suggested that these extracts may be used for the development of food supplements or biofunctional foods that would protect from diseases caused by oxidative stress-induced endothelium damage.

## Figures and Tables

**Table 1:** The sequence of primers used for the assessment of mRNA levels of *NFE2L2*, *GCLC*, *GSR*, *GPX1*, *HMOX1*, *CAT*, *NQO1*, *SOD1*, and *TXN* genes in EA.hy926 cells by qRT-PCR.

Gene	Access no.	Primer (5′-3′)
*CAT*	847	Forward: CCAGAAGAAAGCGGTCAAGAA
		Reverse: TGGATGTGGCTCCCGTAGTC
*SOD1*	6647	Forward: AGGGCATCA TCAATTTCGAG
		Reverse: GGGCCTCAGACTACATCCAA
*TXN*	7295	Forward: TTTCCATCGGTCCTTACAGC
		Reverse: TTGGCTCCAGAAAATTCACC
*HMOX1*	3162	Forward: GGCCTGGCCTTCTTCACCTT
		Reverse: GAGGGGCTCTGGTCCTTGGT
*NFE2L2*	4780	Forward: *ATTGCCTGTAAGTCCTGGTCA*
		*Reverse: ACTGCTCTTTGGACATCATTTCG*
*NQO1*	1728	Forward: GGGCAAGTCCATCCCAACTG
		Reverse: GCAAGTCAGGGAAGCCTGGA
*GCLC*	2729	Forward: GAAGAAGATATTTTTCCTGTCATTGAT
		Reverse: CCATTCATGTATTGAAGAGTGAATTT
*GSR*	2936	Forward: CCAGCTTAGGAATAACCAGCGATGG
		Reverse: GTCTTTTTAACCTCCTTGACCTGGGAGAAC
*GPX1*	2876	Forward: CGCTTCCAGACCATTGACATC
		Reverse: CGAGGTGGTATTTTCTGTAAGATCA
*GAPDH*	2597	*Forward: TGCACCACCAACTGCTTAG*
		*Reverse: GATGCAGGGATGATGTTC*

**Table 2:** Total phenolic content, free radical scavenging activity against ABTS^•+^ and O_2_^•−^ radicals, protective activity against peroxyl (ROO•) radical-induced DNA damage, and reducing power of the extracts.

Plant variety	TPC^a^ (mg GAE/gr dw)	IC_50_ (*μ*g/ml)^e^			
		ABTS^•+^	O_2_^•−^	ROO•	Reducing power^e^ (RP_0.5AU_)^d^
*Carthamus lanatus*	408	7.9 ± 0.9^b^	6.3 ± 0.4^b^	110.0 ± 8.2^c^	5.0 ± 0.3^b^
*Cichorium intybus*	320	9.1 ± 0.6	8.2 ± 0.7	105.0 ± 7.6	6.0 ± 0.2
*Cichorium spinosum*	117	28.0 ± 3.1	21.0 ± 1.4	300.0 ± 17.3	8.0 ± 0.6
*Crepis sancta*	288	12.0 ± 1.5	7.5 ± 0.6	132.0 ± 9.8	10.5 ± 0.9
*Sonchus asper*	56	66.0 ± 7.2	56.0 ± 3.8	970.0 ± 53.4	47.0 ± 3.3
*Amaranthus blitum*	63	72.0 ± 9.6	21.0 ± 1.2	443.0 ± 19.5	65.0 ± 4.6

^a^TPC: total polyphenolic content. ^b^Values are the mean ± SD of at least two separate triplicate experiments. ^c^Values are the mean ± SD from three independent experiments. ^d^RP_0.5AU_: extract concentration (*μ*g/ml) caused absorbance of 0.5 at 700 nm. ^e^Values are statistically significant, *p* < 0.05.

## Data Availability

The data used to support the findings of this study are available from the corresponding author upon request.

## Supplementary Materials

Supplementary Materials Values of retention time and area (%) of the relative quantification of main secondary metabolites detected in the extracts by using HPLC-PDA analysis.
