# Supplementary material for: Extracts from the Mediterranean Food Plants Carthamus lanatus, Cichorium intybus, and Cichorium spinosum Enhanced GSH Levels and Increased Nrf2 Expression in Human Endothelial Cells
Source: Oxid Med Cell Longev. 2018 Nov 15;2018:6594101. doi: 10.1155/2018/6594101 (PMC6276477; doi:10.1155/2018/6594101)
Supplement: Supplementary Materials — Values of retention time and area (%) of the relative quantification of main secondary metabolites detected in the extracts by using HPLC-PDA analysis. [file 6594101.f1.pdf]

SUPPLEMENTARY INFORMATION

Relative quantification of main secondary metabolites detected in the chórta decoctions. Detection at 280 nm.

Table 1 Relative quantification of main secondary metabolites in the Cichorium spinosum decoction.

Retention Time (min)	Compound	Area % at 280nm
17.0	caftaric acid	7.7
21.0	caffeoylquinic acid isomer	4.5
26.5	cichoric acid	38.8
29.0-32.0	luteolin diglycoside,<br>quercetin glucuronide,<br>luteolin glucuronide	~ 9.5

Table 2 Relative quantification of main secondary metabolites in the Cichorium intybus decoction.

Retention Time (min)	Compound	Area % at 280nm
17.0	caftaric acid	1.9
21.5	caffeoylquinic acid isomer	6.3
27.0	cichoric acid	50.5
32.0	luteolin glucuronide	14.1
33.8	dicaffeoylquinic acid	2.5

Table 3 Relative quantification of main secondary metabolites in the Crepis sancta decoction.

Retention Time (min)	Compound	Area % at 280nm
17.0	caftaric acid	1.8
21.2	caffeoylquinic acid isomer	9.9
27.0	cichoric acid	53.7
32.0	luteolin glucuronide	9.7
33.9	dicaffeoylquinic acid	2.3

Table 4 Relative quantification of main secondary metabolites in the Sonchus asper decoction.

Retention Time (min)	Compound	Area % at 280nm
17.2	caftaric acid	9.3
21.7	caffeoylquinic acid isomer	2.2
27.3	cichoric acid	28.4
32.0	luteolin glucuronide	16.2
35.0	apigenin glucuronide	8.7

Table 5 Relative quantification of main secondary metabolites in the Carthamus lanatus decoction.

Retention Time (min)	Compound	Area % at 280nm
20.7	caffeoylquinic acid isomer	7.6
29.8	quercetin glucoside	30.8
31.0	luteolin glucoside	27.9
33.5	quercetin acetyl glucoside	15.2
34.9	luteolin acetyl glucoside	7.5

Table 6 Relative quantification of main secondary metabolites in the Amaranthus blitum decoction.

Retention Time (min)	Compound	Area % at 280nm
13.0-21.0	hydroxycinnamates	~ 28.0
25.8	rutin	23.0
29.3	luteolin diglycoside	6.2
47.5, 52.0	triterpene saponins	~ 22.0
